# Evaluation of *Dittrichia viscosa* (L.) Greuter Dried Biomass for Weed Management

**DOI:** 10.3390/plants10010147

**Published:** 2021-01-12

**Authors:** Angela Boari, Maurizio Vurro, Generosa Jenny Calabrese, Mohamed Nesma Zakaria Mahmoud, Eugenio Cazzato, Mariano Fracchiolla

**Affiliations:** 1 Institute of Sciences of Food Production (ISPA), National Research Council (CNR), via Amendola 122/O, 70125 Bari, Italy; angela.boari@ispa.cnr.it (A.B.); maurizio.vurro@ispa.cnr.it (M.V.); 2 International Center for Advanced Mediterranean Agronomic Studies, Mediterranean Agronomic Institute of Bari (CIHEAM Bari), via Ceglie 9, 70010 Valenzano, Italy; calabrese@iamb.it (G.J.C.); nesma.z.mahmoud@agr.cu.edu.eg (M.N.Z.M.); 3 Department of Agricultural and Environmental Science, University of Bari, Via Amendola, 165/A, 70126 Bari, Italy; mariano.fracchiolla@uniba.it

**Keywords:** weeds, sustainable weed management, bioherbicide, allelopathy

## Abstract

*Dittrichia viscosa* (L.) Greuter, a plant species common in the Mediterranean basin, produces several bioactive compounds, some of which have herbicidal effects. A number of greenhouse and field experiments were carried out in order to evaluate if these effects could be obtained also by using the whole plant biomass, to identify the efficacious doses, determine their effects on seed germination and weed emergence, and to evaluate influence of soil characteristics on biomass efficacy. The experiments carried out evidenced that: (i) the dried biomass completely hampers plant emergence when high doses (30–40 kg biomass m^−3^ of soil) are mixed into the soil, or delays it at a lower dose (10 kg m^−3^); (ii) the detrimental effects are not affected by soil type. The exploitation of the *D. viscosa* dried biomass appears to be a feasible option in weed management practices and its potential is discussed.

## 1. Introduction

Among the bio-constraints affecting modern agriculture, weeds represent one of the major causes of crop losses, and their management is one of the most troublesome, expensive and labor-consuming of the agricultural practices. Despite the progress in many technological fields, most of the weed management practices are still based on the use of synthetic chemicals, although two trends are now jeopardizing their use: i) many active ingredients have already been withdrawn for regulatory reasons because of toxicology issues; ii) some active ingredients are becoming less effective as the result of rapid evolution of herbicide-resistant weeds [1]. These issues have led to a renewed interest in the development of biological alternatives to synthetic herbicides. 

Among the possible approaches, the exploitation of plant allelopathy, meant as the ability of plants to produce and release secondary metabolites (allelochemicals) acting as herbicides, has received increased attention in recent decades [2,3,4,5]. Many studies have explored the weed control potential of fresh or dried plants (both crops or weeds) applied to the soil surface (mulching) or incorporated into the soil, able to release compounds inhibiting weed seed germination and plant growth [6,7,8,9,10], although often with controversial or minimal results. The effects of dried biomasses have been investigated by several authors. Among others, Vidotto et al. [11] found effectiveness of several species using powdered dried leaves (2 t ha^−1^) of Jerusalem artichoke (*Helianthus tuberosus* L. Lin et al. [12] found that 100 or 150 g m^−2^ of dried biomass of maidong (*Ophiopogon japonicus* (Thunb.) Ker–Gawler) had inhibitory effects on germination of barnyard grass, (*Echinochloa crusgalli* L.), monchoria (*Monocharia vaginalis* P.) and smallflower umbrella (*Cyperus difformis* L.). Khanh et al. [13] studied Chinese taro (*Alocasia cucullata* (Lour.) G. Don), Jerusalem artichoke (*H. tuberosus*), oleander (*Nerium oleander* L.), passion flower (*Passiflora incarnata* L.), Japanese pagoda tree (*Sophora japonica* L.) and stylo (*Stylosanthes guianensis* (Aublet) Sw.). The application of 1.5 t ha^−1^ of dried biomass of all the tested species reduced weed plant growth and the dry weight of weeds by 60–100% and 70–100%, respectively. De Mastro et al. [14] found that 3.5 kg m^−2^ of dried biomass of an oregano hybrid (*Origanum vulgare* L. ssp. *virilidum* x *O. vulgare* L. ssp. *hirtum* (Link) Iestwart), inhibited the emergence of *Amaranthus graecizans* L. and *Portulaca oleracea* L. in tomato. 

Many weed species have also been tested for the production and release of allelochemicals, such as *Chenopodium album* L. (common lambsquarters), *Medicago denticulata* Willd. (*California burclover*), *Melilotus indica* L. (sweet clover), *Convolvulus arvensis* L. (field bindweed), among others, to control other weed species [15], as *Echinochloa crus-galli* (L.) P. Beauv. (barnyard grass), *Agropyron smithii* Rydb. (western wheatgrass), or *Bromus marginatus* Steud. (mountaine brome) [16]. 

Very often those allelopathic effects are exploited by using the aqueous or sometimes organic extracts from different plant parts (leaves, roots, seeds), with very limited or no knowledge of the chemical composition of the allelopathic fractions, or of the content in bioactive compounds. For example, aqueous extracts of rushfoil (*Croton bonplandianum* Baill.) showed herbicidal potential against weeds such as *Melilotus alba* L. (honey clover), *Vicia sativa* L. (common vetch) and *Medicago hispida* Gaertn. (California burclover) [17]. In another study, the extracts of two weed species, i.e., *E. crus-galli* and *Withania somnifera* (L.) Dunal (winter cherry), were tested for their potential to inhibit germination and seedling growth of *Avena fatua* L. (common wild oat) [18]. 

*Dittrichia viscosa* (L.) Greuter is a plant species common in the Mediterranean Basin, widespread in southern Europe, in the Middle East and in northern Africa. Due to its ecological properties (e.g., allelopathic effects against other plants, production of strong-smelling oil, resistance to predators and diseases, attractiveness for pollinators), the plant has been extensively studied in relation to the production of bioactive compounds. Some of the compounds produced by *D. viscosa* have phytotoxic and allelopathic effects causing inhibition of seed germination and plant growth, or necrosis of leaves [19,20,21]. 

Despite the large number of bioactive metabolites, isolated and chemically and biologically characterized [22,23,24,25] and references therein cited], and the numerous studies carried out by using plant extracts [25,26] the plant has never been the subject of advanced and focused studies aiming to exploit the potential of its biomass for direct use in weed management practices. Thus, it seems of interest to investigate, in a more comprehensive way, the herbicidal effects of *D. viscosa* dried biomass (DB), and in particular, its ability to inhibit weed seed germination, to slow down or hamper plant emergence, to be effective in different soils, and thus finally to be used in integrated weed management.

## 2. Results

### 2.1. Experiment 1: Effects of DB on Emergence of Cress and Wheat

**Figure 1:**
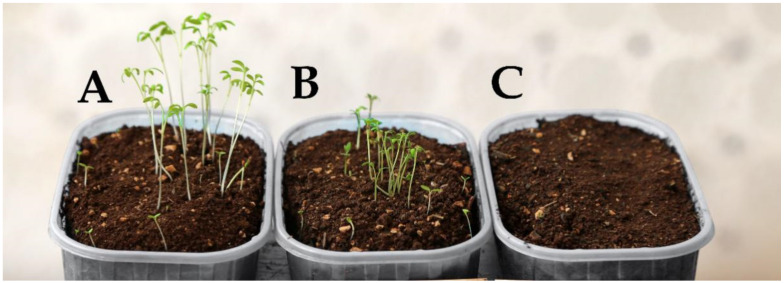
Effect of *Dittrichia viscosa* dried biomass (DB) on the emergence of *Lepidium sativum*, 16 days after sowing. (**A**) control (D0 treatment = no DB added to the soil); (**B**) D10 treatment (10 kg DB m^−3^ of soil); (**C**) D40 treatment (40 kg DB m^−3^ of soil).

As shown in Table 1, 7 DAS (days after sowing) there were no emerged plantlets of cress in D40 and D20 treatments, whereas in D2, D4 and D10 treatments only a few emerged (around 5–6), significantly fewer than in the control D0 (11 on average). At 16 DAS, the emergence was still null in D40, whereas in D20 the number of emerged plantlets increased to 10.5; however, this was statistically lower than the control and the other treatments (Table 1; Figure 1). The lowest D.W. of the emerged plantlets was in D20, although also in D2, D4 and D10 it was significantly lower than in the control.

For durum wheat, 7 DAS there were no emerged plantlets in D20 and D40 treatments. In all the other treatments, the plantlet emergence was significantly lower than the control, ranging between 2.7 to 8.7, against 14.5 for the control. Twelve DAS, only the number of emerged plantlets in D40 (6.2) was statistically lower than the number in the control (17.0). At the end of the experiment, the lowest D.W. was recorded for D40, significantly lower than in the other treatments. Moreover, in all treatments D.W was significantly lower than in the untreated control (D0).

### 2.2. Experiment 2: Effects on Emergence of the Natural Seed Bank in Greenhouse Conditions

The natural infestation of the two soils used for the trials proved to be composed of seven species (Table 2). *Amaranthus retroflexus* L., *C. album*, *Lamium amplexicaule* L. and *Urtica dioica* L. emerged significantly less in D30 and D20 treatments than in the other trays. In D10, the emergence was lower than in the untreated trays. As for *Calendula arvensis* (Vaill.) L. and *Veronica hederifolia* L., all the treated trays showed a statistically lower number of emerged plantlets with respect to the untreated control (D0). The emergence of *Solanum nigrum* L. was the lowest in D30 although statistically not different from D20 that, in turn, was not different from D10. Statistically higher emergence was recorded in the control. The total dry weight determined for both soils was lower in treated than in untreated trays, although data were not statistically different (data not reported).

### 2.3. Experiment 3: Effects on Emergence of the Natural Seed Bank in Open Field Conditions

**Figure 2:**
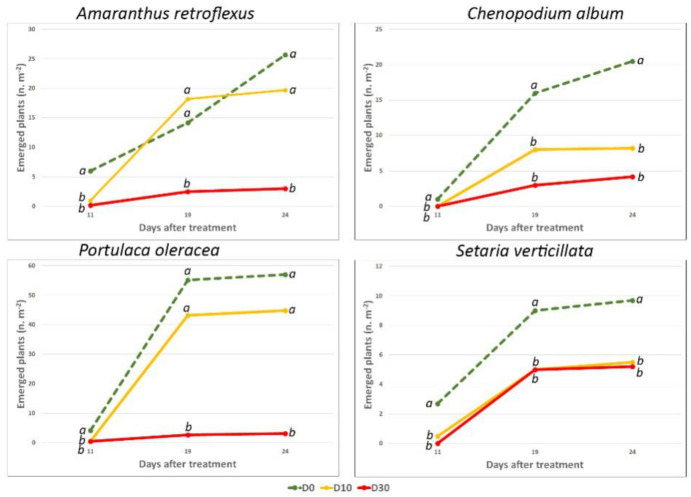
Effects of DB on the emergence of natural infestation. For each time of the survey (days after treatment), data followed by different letters are significantly different at 0.05 P (Duncan’s test).

**Figure 3:**
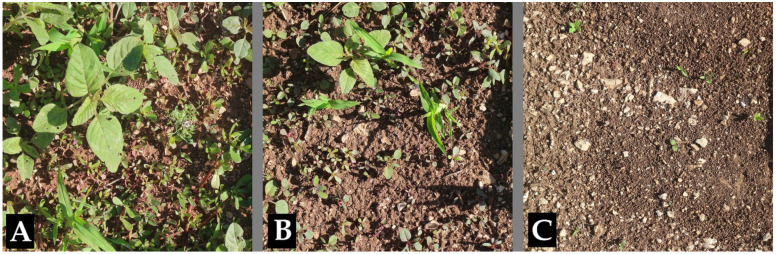
Effect of *Dittrichia viscosa* dried biomass (DB) on the emergence of natural seed bank in the field experiment, 19 days after treatment. (**A**) control (D0 treatment = no DB added to the soil); (**B**) D10 treatment (10 kg DB m^−3^ of soil for a layer of 0.04 m); (**C**) D30 treatment (30 kg DB m^−3^ of soil for a layer of 0.04 m).

In the course of the open field experiment, four species emerged, all typical of the period (May-June), i.e.: *A. retroflexus* L. (redroot pigweed), *C. album* (common lambsquarters), *P. oleracea* L. (common purslane), *Setaria verticillata* (Vill.) L. (hooked bristlegrass). The quite small size of the plots resulted in a rather uniform infestation. With respect to the control, 11 DAT the emergence of *A. retroflexus* and *P. oleracea* was significantly lower both in D10 and in D30. Conversely, 19 and 24 DAT, only in D30 plots was the emergence lower than in the D0 (Figure 2 and Figure 3). The number of the emerged plants of *C. album* was statistically lower in treated plots than in the control at all times. Concerning *S. verticillata*, the number of emerged plants was lower in D10 and D30 than in D0 only 11 DAT; in the other surveys, although fewer plants emerged in the treated plots, differences were not significant. At the end of the experiment (24 DAT) the total aerial F.W. in the treated plots (0.18 and 0.11 kg m^−2^, D10 and D30, respectively) was much less than in the control (0.7 kg m^−2^).

### 2.4. Experiment 4: Effects on Seedlings

Two weeks after the burial of the germinated seeds (Table 3), in D30 treatment the number of emerged seedlings (4.0) was significantly lower than in the control (16.0). A slightly higher number of seedlings was observed in D20 (4.7) compared to D30, but still statistically different from the control. In D10 treatment, the number of emerged plantlets was lower than in the control (14.0) but not significantly different.

### 2.5. Experiment 5: Reversibility of the Effects on Seed Germination

As shown in Table 4, no plants emerged in the trays of the treatment D30 + D30 up to the 10th day after reburial. For all surveys, a number of plants statistically lower than in D0 is reported for the D30 + D0 treatment. Meanwhile, emerged plants in D30 + D0 were lower than in the control only 2–4 and 7 days after reburial.

### 2.6. Experiment 6: DB Efficacy in Relation to Different Soil Texture

The three typologies of soils and their interaction with the doses of DB were not statistically relevant. On average and in line with expectations, soils treated with DB appear to affect the emergence of plants and show the lowest number of emerged plants for D30 treatment, but results do not differ statistically from D20 (data not reported).

## 3. Discussion

Scientific literature reports that plant extracts of *D. viscosa* have effects both on seeds’ germination and on seedling growth. The inhibitory effect of this plant is probably due to the combined effects of many secondary metabolites contained in the plant tissues, such as flavonoids, sesquiterpene lactones, sesquiterpene acids, triterpenoids and caffeic acids [25]. The leaf exudates of *D. viscosa* show an inhibitory effect on the germination of lettuce seeds and delay the germination of *Malcolmia maritima* (L.) W.T. Aiton seeds [26]. The use of *D. viscosa* extracts from leaves and flowers (but not from stems and roots) caused a reduction of the germinability of *Raphanus sativus* L., *Lactuca sativa* L., *Silybum marianum* (L.) Gaertn. and *Peganum harmala* L. When the residues of the plant were incorporated into the soil, the reduction in root and shoot length of the target species ranged between 34% and 100%. Inuloxins are able to inhibit or to stimulate the germination of seeds of parasitic weeds [18,19]. The use of aqueous extracts from leaves and flowers to irrigate the soil reduced the length of seedlings by 100% for Peganum and 82% for Silybum [27]. Moreover, Dor and Hershenhorn [21] found that ground dry leaves mixed at a dose of 10 g kg^−1^ soil did not inhibit seed germination or development of tested plants (*Malva parviflora* L., *Avena sterilis* L., *Beta vulgaris* L., *Lycopersicon esculentum* Mill., *Triticum durum* Desf. and *Gossypium hirsutum* L.). Only treatments with higher doses (100 g kg^−1^ soil) significantly inhibited the growth of the tested weeds. *B. vulgaris* and *A. sterilis* were less sensitive, with biomass decreasing to about 65% of controls.

Many of these studies considered the herbicidal efficacy of single metabolites, or of aqueous extracts, on a limited number of weeds. Our study aimed at testing the plant biomass, thus containing the whole set of bioactive metabolites, against a larger number of weeds, in order to have more comprehensive results about its effectiveness against weeds. DB effectiveness on cress was observed from a dose of 20 kg of DB m^−3^ of soil, whereas all doses below caused only a delay of germination. The highest dose used (40 kg m^−3^) completely hampered emergence, at least for the duration of the experiment (16 days). With regards to the effects on germinated seeds (seedlings), only the higher doses (20 and 30 kg m^−3^) had an inhibitory effect on their growth. Moreover, the characteristics of the soils seem to have no influence on the activity. The same trend was observed with the species belonging to the natural seed bank, even if slight differences were observed depending on the species. The field experiment confirmed the same results obtained in the greenhouse experiments: hooked bristlegrass, a species belonging to the *Poaceae* family like wheat, was less affected than other species. This result is in agreement with that of Dor and Hershenhorn [21], who reported that *A. sterilis* was less sensitive than other species, confirming that grass species are more resistant to the allelopathic compounds contained or released by *D. viscosa* DB.

The allelopathic effects on seed germination or plant growth due to plant exudates or tissues degradation are well known also for cover crops (see, for example, [28,29]). Similar inhibitory effects were recorded, although the amount of biomass that showed acceptable effectiveness was much higher than that left in the soil by a cover crop; we can estimate that an acceptable effect can be reached with doses ranging between 10 and 30 kg m^−3^ of soil. Based on our findings, we suppose that biomass deriving from *D. viscosa* could be a valuable material to be used as “amendment with herbicidal activity”. The dried biomass preserved its efficacy for a longer time when stored dry; this could ease its practical use.

It is also interesting to note that the inhibition of the germination of cress seeds was reversible. Indeed, in presence of high biomass content (30 kg m^−3^), the germination was inhibited; however, once the seeds were transferred to the untreated soil, the germination occurred at the same rate as in the control. This finding leads us to suppose that compounds released by the biomass do not kill the seeds and, as soon as they are degraded, the germination process restarts. 

Although the lower doses have only a delaying effect on germination, this property could be exploited in many crops to keep the field free of weeds only during the critical period of weed interference [30]. This would be particularly important in minor crops, where the scarce availability or absence of registered herbicides urge the integrated use of nonchemical or alternative weed control methods [31]. Given the considerable dose at which *D. viscosa* biomass is effective, other application strategies could be investigated and adopted to reduce the amount of the biomass necessary to be effective in weed management. For example, the incorporation of the biomass only in-row could be effectively combined with mechanical or physical interventions, more easily adoptable intra-row. The approach of incorporating the biomass only in-row was adopted, for example, by De Mastro et al. [14] in transplanted tomato, and allowed reduction of up to 50% of the dose of oregano hybrid with high content of carvacrol necessary for controlling weeds (*A. graecizans, P. oleracea*).

To make this hypothesis transferable to the field, many other aspects need to be investigated, e.g., the selectivity of the biomass toward weed species, the sensitivity of crop species, the efficacy over time and the application methods by devoting attention to the reduction of the doses.

## 4. Materials and Methods

### 4.1. Location and Site of the Experiments

The greenhouse experiments were performed at the Weed Science Laboratory and Greenhouse of the Department of Agricultural and Environmental Science at the University of Bari (41.111° N; 16.881° E) from autumn 2019 to winter/spring 2020. The open field experiment was performed in a field in Noicattaro (Southern Italy, 41.051° N; 16.983° E).

### 4.2. Dried Biomass (DB)

In late spring and summer of 2019, fresh stems were harvested from *D. viscosa* plants growing in rural areas, roadsides and field margins in Bari. The plant material was dried in a fan oven at 40 °C for two days and then ground using a lab mill (Cutter Mixer K35) after removal of the woody parts and reduced into a powder (particles having a size below 1 mm). The resulting dried biomass (DB) was homogenized in order to obtain the material to be used in the whole set of experiments. Approximately 60 kg of fresh plants were processed and around 16.3 kg of DB were obtained. The material was stored, in the dark, in plastic bags at temperature between 15 and 20 °C. 

### 4.3. Indicator Plant Species

Cress (*Lepidium sativum* L.) and durum wheat (*Triticum durum* Desf.) were used as indicator plant species to evaluate the herbicidal effects, because their seeds germinate quickly and uniformly at a high rate with rapid growth. Indeed, seeds collected from wild plants are often affected by dormancy, low germination rate and high genetic variability. Therefore, we considered wild-harvested seeds unsuitable for our assays. The emergence of the natural seed bank from the soils was considered in some experiments (see above), in order to have more information directly related to the possible practical use of the DB. 

### 4.4. Preparation of the Substrate Necessary for Seed Germination or Plant Growth 

Soil used for all the greenhouse tests had a content of 38% of sand, 28% of silt and 34% of clay; the organic matter was 1.5%, pH was 7.5 and active calcium was 24.8 g kg^−1^. Soil was sieved to remove debris, stones or other undesired particles, and mixed throughout to obtain a homogeneous material. DB was mixed into the soil at the defined amounts (see above), and the mixture used to fill trays. Soil in the trays was daily watered in order to keep the soil continuously wet without exceeding field capacity to avoid leaching of water and solutes, to enable seed germination and plant growth. 

### 4.5. Experiments

#### 4.5.1. Experiment 1: Effects of DB on Emergence of Indicator Plants

This experiment was performed to achieve information about dose effectiveness. Twenty seeds of each tested species were sown in trays (size 0.2 × 0.11 × 0.085 m, filled with 0.0015 m^3^ of soil) with 5 DB amounts (2, 4, 10, 20, 40 kg m^3^ of soil). Preliminary tests carried out with the biomass of other plant species have agreed to exclude that the inhibitory effect of *D. viscosa* biomass was simply due to physical interference (unpublished). Thus, we did not prepare a control with possible inert material, but only control trays with no DB in the soil.

The number of germinated seeds in each tray was recorded twice: the first time when 50% of seeds emerged in the control; the second time when no new emerging plants were recorded in the control for two consecutive days. At the end of the experiment, aerial fresh (F.W.) and dry weight (D.W.) was measured.

#### 4.5.2. Experiment 2: Effects of DB on Emergence of the Natural Seed Bank in Greenhouse Conditions

This experiment was carried out by using two soil types collected from two different fields and having different seed banks. The first one (hereinafter named soil 1) was a clay loam soil with pH = 7.5 and having 1.5% of organic matter. The second one (hereinafter named soil 2) was a sandy loam soil with pH = 8.4 and having 1.9% of organic matter. Trays (0.2 × 0.26 × 0.1 m) were filled with 0.004 m^3^ of soil mixed with 0, 10, 20, 30 DB kg m^3^ soil. At the end of the experiment (25 days after soil preparation), the emerged plant species were identified and counted for each tray, and total aerial F.W. and D.W. assessed.

#### 4.5.3. Experiment 3: Effects on Emergence of the Natural Seed Bank in Open Field Conditions

This experiment was carried out from May and June 2020 and aimed to evaluate the effects of the DB on natural infestation in field conditions. For this purpose, 0.01 m^3^ of soil corresponding to 0.04 m depth was removed from the surface of square plots (0.5 m by side). Soil was mixed with 10 or 30 kg m^−3^ of biomass and distributed back uniformly over the plots. No-treated plots were also included in the experiment; also in these plots, soil was removed and distributed back, to create the same conditions as in the treated plots. Plots were leveled, smoothed out and watered one time at the beginning of the test. Natural infestation was recorded 11, 19 and 24 days after treatment (DAT) by placing a squared metal frame (0.25 m side) in the middle of the plot and counting the emerged plants for each botanical species. At the end of the experiments (24 DAT), the aerial parts of the plants were harvested by cutting and the total aerial F.W. was finally determined. 

#### 4.5.4. Experiment 4: Effects on Seedlings 

This experiment aimed to evaluate the effects of DB on seeds immediately after germination (i.e., after appearance of radicle and hypocotyl). Under laboratory conditions, cress seeds were first rinsed with tap water and then placed on filter papers Whatman^®^ (thirty per each), placing each filter in a Petri dish with a diameter of 90 mm. Sixteen Petri dishes were prepared and stored for two days in a growth cabinet, at 25 °C in the dark, to allow seed germination. Then, the seedlings were transferred to the greenhouse, and placed in trays (0.07 × 0.13 × 0.005 m) containing 0.0004 m^3^ of soil mixed with different DB amounts (0, 10, 20, and 30 kg m^−3^ of soil). The seedlings were placed on the soil surface by using tweezers and covered with a thin layer of soil. After two weeks, the number of emerged seedlings was counted. 

#### 4.5.5. Experiment 5: Reversibility of the Effects on Seed Germination

After having assessed the effects on seeds immediately after germination (experiment 4), this experiment was performed to evaluate the ability of seeds to germinate (in a no-treated soil) after they were kept in a treated soil in which germination was inhibited; more clearly, the reversibility of the DB effects on seed germination was evaluated. In this test, 20 seeds were placed in a tea bag-like sachet and buried in trays (0.07 × 0.13 × 0.05 m) filled with 0.0004 m^3^ of soil mixed with 30 kg m^−3^ (D30, that is a dose that completely inhibited the germination), or no biomass (D0, in order to allow germination), for 4 days. Once exhumed, they were buried again in soil (8th day of test), with or without DB according to the following treatments:

- Treatment 1: D0 + D0 (i.e., control) = seeds buried in untreated soil (with no biomass) were then buried again in untreated soil.
- Treatment 2: D30 + D0 (i.e., inhibitory factor removed) = seeds previously buried in treated soil were buried in untreated soil to remove the inhibitory factor.
- Treatment 3: D30 + D30 (i.e., inhibitory factor not removed) = seeds buried in treated soil were buried again in treated soil.

The emerged plants in each tray were counted at 2, 4, 7 and 10 days after reburial.

#### 4.5.6. Experiment 6: DB Efficacy in Relation to Different Soil Texture

The possible influence of soil type was evaluated in this experiment. Three different types of soil, collected in different fields and characterized by different textures (sand-silt-clay), were used: clay loam (38.0–28.0–34.0%), sandy loam (60.8–27.2–12.0%) and sandy-clay loam (67.7–11.6–20.7%) soil. The content of organic matter differed marginally between the different soils and was respectively: 1.5–1.9% and 1.9%. Fifty seeds of cress were sown in trays (0.2 × 0.26 × 0.1 m) previously filled with increasing DB doses (0, 10, 20, and 30 kg m^−3^ of soil) mixed into the different soils. After 2 weeks, the number of emerged cress seedling was counted.

### 4.6. Experimental Design and Statistical Analysis

For the greenhouse experiments, a completely randomized experimental design was used with the exception of experiment 6, which was performed following a two-way completely randomized design (soils and doses as factors). Four replicates for each treatment were prepared. The field experiment was arranged according to the randomized block design with four replications. All the collected data were subjected to analysis of variance and differences among means were compared with Duncan’s test or Student’s *t*-test. The CoStat Statistics Software (www.cohort.com) was used.

## 5. Conclusions

The preliminary results obtained in this study show that the dried biomass of *D. viscosa* affects both seed emergence and plant growth and thus it could be effectively used to prevent weed emergence. Although many studies would be necessary to test the efficacy in different field conditions or farming systems, the use of biomass could be suitable in integrated and sustainable weed management programs, particularly in organic farming, where the use of chemicals is not allowed, or in minor crops, where the number of available registered herbicides is low, or even in small farms that cannot afford the cost of equipment for treatments. Indeed, an advantage of using biomass is that, unlike the plant extracts, it is an easily obtainable material with low technological or energy demands. Moreover, being a flowering Mediterranean plant species of the *Asteraceae* family having high seed production, very dense canopy and great capability to adapt to adverse conditions [32], *D. viscosa* could be grown on-farm, or even as an industrial crop, in order to produce the necessary amounts of biomass.

## Figures and Tables

**Table 1:** Effects of dried biomass (DB) on cress (*Lepidium sativum*) and durum wheat (*Triticum durum*) emergence.

Treatments **	Emerged Plants *					
	*Lepidium sativum*			*Triticum durum*		
	DAS		D.W.	DAS		D.W.
	7	16		7	12	
D0	11.7 a	15.5 a	0.04 a	14.5 a	17.0 a	0.31 a
D2	6.2 b	16.2 a	0.03 b	8.7 b	17.5 a	0.25 b
D4	6.2 b	16.5 a	0.03 b	2.7 c	19.0 a	0.23 b
D10	5.7 b	17.5 a	0.03 b	4.7 bc	18.0 a	0.23 b
D20	0.0 c	10.5 b	0.02 c	0.0 c	19.0 a	0.16 c
D40	0.0 c	0.0 c	0.0 d	0.0 c	6.2 b	0.04 d

* In each column, data followed by different letters are significantly different (P = 0.05, Duncan’s Test). ** The number following “D”, in the treatment code, indicates the dose of DB added to the soil (kg m^−3^).

**Table 2:** Effects of DB on the emergence of natural seed bank of two different soils.

Scientific Name	Common Name	Emerged Plants (n.) * per Each Treatment **			
		D0	D10	D20	D30
*Amaranthus retroflexus* L. ^a^	redroot pigweed	178.5 a	128.2 b	30.7 c	9.2 c
*Calendula arvensis* (Vaill.) L. ^a^	marigold	3.7 a	0.0 b	0.0 b	0.0 b
*Chenopodium album* L. ^a^	common lambsquarters	169.0 a	52.5 b	18.7 c	7.5 c
*Solanum nigrum* L. ^a^	black nightshade	7.2 a	4.0 b	1.2 bc	0.5 c
*Lamium amplexicaule* L. ^b^	henbit	75.7 a	28.2 b	13.5 c	6.5 c
*Veronica hederifolia* L. ^b^	veronica	14.7 a	3.7 b	3.5 b	1.0 b
*Urtica dioica* L. ^b^	nettle	59.5 a	17.0 b	6.5 c	1.5 c

^a^ From soil 1. ^b^ From soil 2. * In each row, data followed by different letters are significantly different (P = 0.05, Duncan’s Test). ** The number following “D”, in the treatment code, indicates the dose of DB added to the soil (kg m^−3^).

**Table 3:** Effects of the DB on the germinated seeds of cress (*Lepidium sativum* L.).

Treatments *	n. of Emerged Plants **
D0	16.0 a
D10	14.0 a
D20	4.7 b
D30	4.0 b

* The number following “D”, in the treatment code, indicates the dose of DB added to the soil (kg m^−3^). ** In each column, data followed by different letters are significantly different (P = 0.05, Duncan’s Test).

**Table 4:** Effects of DB on pre-treated seeds of cress (*Lepidium sativum* L.).

	Emerged Plants (n.) from Reburied Seeds *			
Treatments **	Days After Reburial			
	2	4	7	10
D0 + D0	9.0 a	12.0 a	13.7 a	14.3 a
D30 + D0	4.0 b	8.0 b	9.3 b	13.7 a
D30 + D30	0.0 c	0.0 c	0.0 c	0.0 b

* In each column, data followed by different letters are significantly different (P = 0.05, Duncan’s Test). ** D0 = Control (100% of seeds had germinated at the time of exhumation). D30 + D0 = inhibitory factor removed (no seed had germinated at the time of exhumation). D30 + D30 = inhibitory factor not removed (no seed had germinated at the time of exhumation). The number following “D”, in the treatment code, indicates the dose of DB added to the soil (kg m^−3^).

## Data Availability

Data sharing not applicable.
